# Metabarcoding of protozoa and helminth in black-necked cranes: a high prevalence of parasites and free-living amoebae[Fn FN1]

**DOI:** 10.1051/parasite/2024028

**Published:** 2024-05-30

**Authors:** Mengshi Yu, Wenhao Li, Xin He, Guiwen He, Yonfang Yao, Yuanjian Wang, Mingcui Shao, Tingsong Xiong, Huailiang Xu, Junsong Zhao

**Affiliations:** 1 College of Agronomy and Life Sciences, Zhaotong University Zhaotong 657000 PR China; 2 Yunnan Key Laboratory of Gastrodia and Fungi Symbiotic Biology, Zhaotong University Zhaotong 657000 PR China; 3 College of Life Science, Sichuan Agricultural University Ya’an 625014 PR China; 4 Sichuan Academy of Grassland Sciences Chengdu 610000 PR China; 5 Management Bureau of Dashanbao Black-Necked Crane National Nature Reserve, Yunnan Province Zhaotong 657000 Yunnan PR China

**Keywords:** *Grus nigricollis*, 18S rDNA, Helminth, Free-living amoebae

## Abstract

Parasites and free-living amoebae (FLA) are common pathogens that pose threats to wildlife and humans. The black-necked crane (*Grus nigricollis*) is a near-threatened species and there is a shortage of research on its parasite diversity. Our study aimed to use noninvasive methods to detect intestinal parasites and pathogenic FLA in *G. nigricollis* using high-throughput sequencing (HTS) based on the 18S rDNA V9 region. A total of 38 fresh fecal samples were collected in Dashanbao, China, during the overwintering period (early-, middle I-, middle II-, and late-winter). Based on the 18S data, eight genera of parasites were identified, including three protozoan parasites: *Eimeria* sp. (92.1%) was the dominant parasite, followed by *Tetratrichomonas* sp. (36.8%) and *Theileria* sp. (2.6%). Five genera of helminths were found: *Echinostoma* sp. (100%), *Posthodiplostomum* sp. (50.0%), *Euryhelmis* sp. (26.3%), *Eucoleus* sp. (50.0%), and *Halomonhystera* sp. (2.6%). Additionally, eight genera of FLA were detected, including the known pathogens *Acanthamoeba* spp. (*n* = 13) and *Allovahlkampfia* spp. (*n* = 3). Specific PCRs were used to further identify the species of some parasites and FLA. Furthermore, the 18S data indicated significant changes in the relative abundance and genus diversity of the protozoan parasites and FLA among the four periods. These results underscore the importance of long-term monitoring of pathogens in black-necked cranes to protect this near-endangered species.

## Abstract (fr)

Les parasites et les amibes libres sont des agents pathogènes courants qui constituent une menace pour la faune et les humains. La grue à cou noir (*Grus nigricollis*) est une espèce quasi menacée et les recherches sur sa diversité parasitaire sont insuffisantes. Notre étude visait à utiliser des méthodes non invasives pour détecter les parasites intestinaux et les amibes libres pathogènes chez *G. nigricollis* en utilisant le séquençage à haut débit basé sur la région V9 de l’ADNr 18S. Au total, 38 échantillons de matières fécales fraîches ont été collectés à Dashanbao, en Chine, au cours de la période d’hivernage (début, milieu I, milieu II et fin de l’hiver). Sur la base des données 18S, huit genres de parasites ont été identifiés, dont trois parasites protozoaires : *Eimeria* sp. (92,1 %) était le parasite dominant, suivi de *Tetratrichomonas* sp. (36,8 %) et *Theileria* sp. (2,6 %). Cinq genres d’helminthes ont été trouvés : *Echinostoma* sp. (100 %), *Posthodiplostomum* sp. (50,0 %), *Euryhelmis* sp. (26,3 %), *Eucoleus* sp. (50,0 %) et *Halomonhystera* sp. (2,6 %). De plus, huit genres d’amibes libres ont été détectés, y compris les agents pathogènes connus *Acanthamoeba* spp. (*n* = 13) et *Allovahlkampfia* spp. (*n* = 3). Des PCR spécifiques ont été utilisées pour identifier davantage les espèces de certains parasites et amibes libres. En outre, les données 18S ont indiqué des changements significatifs dans l’abondance relative et la diversité des genres des parasites protozoaires et des amibes au cours des quatre périodes. Ces résultats soulignent l’importance de la surveillance à long terme des agents pathogènes chez les grues à cou noir pour protéger cette espèce quasi menacée.

## Introduction

The black-necked crane (*Grus nigricollis*) is the only alpine crane species. It is mainly distributed in China, India, Bhutan, and adjacent areas, and its population has been estimated at 17,389–17,610 individuals in total [14]. The black-necked crane is classified as a Near Threatened species by the International Union for Conservation of Nature (IUCN) and listed as a Class I national key protected wildlife in China. Furthermore, its major breeding and wintering areas in China are located on the Qinghai-Tibet and Yunnan-Guizhou Plateaus, and most of the areas have been designated as nature reserves [57]. In China, the black-necked crane is one of the bird species zoologists focus on, and scientific studies on it have been intensive. However, the studies on it are mainly concentrated on ecology, such as migration routes [53, 72], and habitat characteristics [36, 42, 76]. To date, there is a shortage of research on the pathogen detection of the black-necked crane, because it is difficult to obtain samples such as blood samples or intestinal contents for pathogen detection, and the invasive sampling method is usually not allowed as it may cause damage to this near-threatened species.

In zoological research, collecting the feces of wild animals from the environment is an important way to obtain biological information. This noninvasive sampling method is of great importance for the study on endangered animals and has been widely used for pathogen detection, especially intestinal parasites. Previous studies investigated the prevalence of eimeriid coccidia [41] and helminths [45] in the feces of black-necked cranes. Nevertheless, these studies relied on morphological identification and only focused on specific parasite species. Compared to molecular methods, the microscopic examination has lower sensitivity and specificity [75]. However, molecular methods that targeted enrichment to detect the DNA of specific parasites from fecal samples make it difficult to obtain a complete picture of the parasite composition in the host [8]. At present, high-throughput sequencing (HTS) is an established practice for detecting microorganisms and characterizing their diversity in various samples [4, 8, 31]. The HTS has been used to analyze the gut microbiota community of the black-necked crane [40, 79], but these studies had a narrow taxonomic focus, which was the symbiotic bacteria. The 18S rRNA gene is the most common marker used for eukaryotic microorganisms [23, 54], and the HTS based on the 18S rDNA is a recognized and well-established method for detecting and characterizing parasite diversity in the host [34, 73]. Therefore, our study used this method to obtain data on eukaryotic microorganism diversity in black-necked crane fecal samples. We aimed to investigate protozoa and helminth composition and further identify parasite diversity in black-necked cranes.

Fecal samples of water birds can be used not only for parasite detection, but also to provide information on the composition of environmental microbiota [5]. Free-living amoebae (FLA) are protozoa widely distributed in the environment; some genera are known to be pathogens, causing amoebic encephalitis in humans, and are becoming a challenge for public health [50]. Meanwhile, FLA are not only facultative pathogens, but can also be vehicles for pathogenic bacteria [50], which makes FLA microorganisms of increased concerned. Black-necked cranes mainly inhabit meadows and wetlands and usually feed on plant roots, insects, and fish [19, 42], which may lead to exposure to FLA in the soil and water. Although the impact of FLA on the birds is unknown, detecting FLA through feces can indirectly reflect potential public health risks. Therefore, in addition to parasites, we also aimed to identify FLA in fecal samples of black-necked cranes.

In this study, we characterized the composition of protozoa and helminths in fecal samples of the wintering black-necked crane in the Dashanbao Wetland Nature Reserve during the overwintering period. Our research provides new data on parasite diversity in black-necked cranes and is of great importance for conservation.

## Methods

### Study area and sample collection

The Yunnan Dashanbao Nature Reserve (27° 18^′^ 38^″^–27° 28^′^ 42^″^ N, 103° 14^′^ 55^″^–103° 18^′^ 38^″^ E; 3,000–3,200 m above sea level) is located in the southwestern region of China and serves as a crucial wintering habitat for black-necked cranes. The wintering population migrates to the Zoige Grassland for breeding and summering from late March to early April each year and returns for wintering in December [72]. A total of 38 fresh fecal samples of the black-necked cranes were collected in the Dashanbao Nature Reserve during the overwintering period (December 2021–March 2022). According to the wintering process of black-necked cranes, four periods were further divided: December is the early wintering period (Early, *n* = 10), January is the middle I wintering period (Middle I, *n* = 10), February is the middle II wintering period (Middle II, *n* = 9), and March is the late wintering period (Late, *n* = 9). Collection of fecal samples was opportunistic; we continuously observed black-necked cranes in a day and collected samples using sterile gloves immediately after we found them defecating. To largely avoid the repeated collection of samples from the same individual, we implemented a sampling strategy with intervals greater than 5 m. Fresh fecal samples were collected in sterile bags and immediately stored in an ice box. Within 2 h, the samples were transported to the laboratory and stored at −80 °C until DNA extraction. To prevent contamination from the external environment, the outer part of the feces was removed before DNA extraction, and only the inner part was used for the formal experiment.

### DNA extraction and PCR amplification

Total fecal DNA extraction was performed using a QIAamp DNA stool minikit (QIAGEN, Inc., Valencia, CA, United States), following the manufacturer’s guidelines. Extracted DNA was quantified and evaluated for purity using Qubit 2.0 Fluorometer (Life Technologies, Carlsbad, CA, USA) and gel electrophoresis, respectively. An ~260 bp region of the 18S rDNA V9 hypervariable region was PCR-amplified using the following primers: 1391f (5^′^-GTACACACCGCCCGTC-3^′^) and EukBr (5^′^-TGATCCTTCTGCAGGTTCACCTAC-3^′^) [34]. PCR was conducted in a 25 μL reaction volume containing 10 ng of template DNA, 0.5 μM of each primer, and 12.5 units of Q5 Hot Start High-Fidelity 2X Master Mix (New England Biolabs: Ipswich, MA, United States). The cycling conditions were initial denaturation at 98 °C for 1 min; followed by 30 cycles at 98 °C for 10 s, 50 °C for 30 s, and 72 °C for 30 s; and final extension at 72 °C for 5 min. The PCR products were pooled in equimolar concentrations on a 2% agarose gel, and purified PCR products were sequenced using the Illumina NovaSeq 6,000 platform PE250 (Illumina, San Diego, CA, United States) at Novogene (Beijing, China).

### Bioinformatics

The barcode and primers of the raw data were removed using the plug-in “cutadapt” [33] of QIIME 2, and the plug-in “DADA2” was then employed for quality control, including removal of sequences containing Ns, sequences shorter than 50 bp, and low-quality sequences [7]. Subsequently, the paired-end sequences were merged and chimeras were removed to obtain amplicon sequence variants (ASVs). The naive Bayesian classifier method was implemented for taxonomic assignment of ASVs based on the SILVA v128 database [54]. To minimize the difference in sequencing depth across samples, data were rarefied to a sampling depth of 52,034 reads per sample for the downstream analysis. Finally, we removed every ASV that was not assigned to Amoebozoa, Apicomplexa, Apusozoa, Cercozoa, Choanozoa, Ciliophora, Foraminifera, Loukozoa, Metamonada, Percolozoa, and Radiolaria in R [67]; then, we manually investigated taxa belonging to protozoa. We also manually investigated the helminth taxonomic groups.

### Statistical analysis

The R package “vegan” [18] was used to calculate the alpha diversity and to perform PERMANOVA analysis. The R package “pairwiseAdonis” [43] was used to perform pairwise PERMANOVA analysis with a Benjamini-Hochberg correction for multiple comparisons. The visualization process was mainly realized using the “ggplot2” [74] and “pheatmap” [35] packages in R [55]. The relative abundance of 11 genera of the protozoan parasites and FLA based on the four overwintering periods was characterized by the online platform [77].

### Specific PCR amplification and sequencing

Among the parasites and FLA identified from the 18S data, *Tetratrichomonas* spp. [22, 39], and *Echinostoma* spp. [47, 48, 66] are common pathogenic parasites in birds and humans. *Acanthamoeba* spp. is also known as the pathogen causing granulomatous amoebic encephalitis in humans [71]. Due to potential pathogenicity to the black-necked crane and humans, we used specific PCR to further identify the species of the three genera. We did not identify *Eimeria* spp., because a previous study had reported the species in black-necked cranes in Dashanbao. We also used the specific PCR to verify the positive result for *Theileria* sp. in one sample [2]. In short, 10 primer pairs (Table S1) were used to detect the species of *Acanthamoeba* sp. [63], *Tetratrichomonas* sp. [22], *Theileria* sp. [2], and *Echinostoma* sp. (including six zoonotic species: *E. revolutum*, *E. ilocanum*, *E. hortense*, *E. trivolvis*, *E. cinetorchis*, and *E. macrorchis*) [47, 48, 66]. The cycling parameters employed for these reactions were described in previous studies.

Each specimen was analyzed twice using 2 μL of extracted DNA per PCR, and 2 × PCRmixture (CoWin Biosciences Company) was used for the PCR amplifications. Then, the PCR products were examined by electrophoresis in 1.5% agarose gels containing ethidium bromide. Finally, all appropriately sized PCR products were sequenced at Tsingke Biotech (Chengdu, China) by performing bidirectional sequencing. The original sequences were edited manually with SeqMan of DNAstar7.0, and further aligned in GenBank using the Basic Local Alignment Search Tool (BLAST) to determine the species.

## Results

### Diversity of protozoa and helminths in *Grus nigricollis*

**Supplementary Figure S1.**
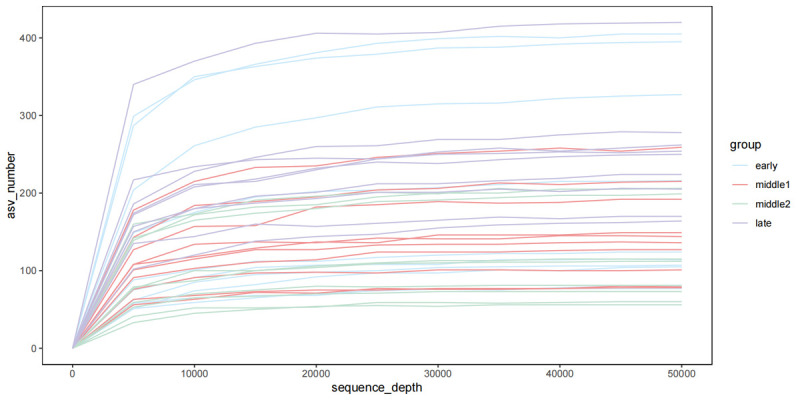
Rarefaction curve of 18S amplicon sequencing.

A total of 3,295,491 (86,723 ± 13,198) raw reads were generated. After quality control and removal of chimeric sequences, a total of 3,013,406 (79,300 ± 11,614) clean reads were retained, accounting for 91.4% of the raw sequences. The results yielded a total of 2,318 (178 ± 97) ASVs, ranging from 56 to 420 ASVs per sample. Rarefaction curves of the number of ASVs with increasing sequence depth of samples (Figure S1), indicating enough sequence depth.

**Figure 1:**
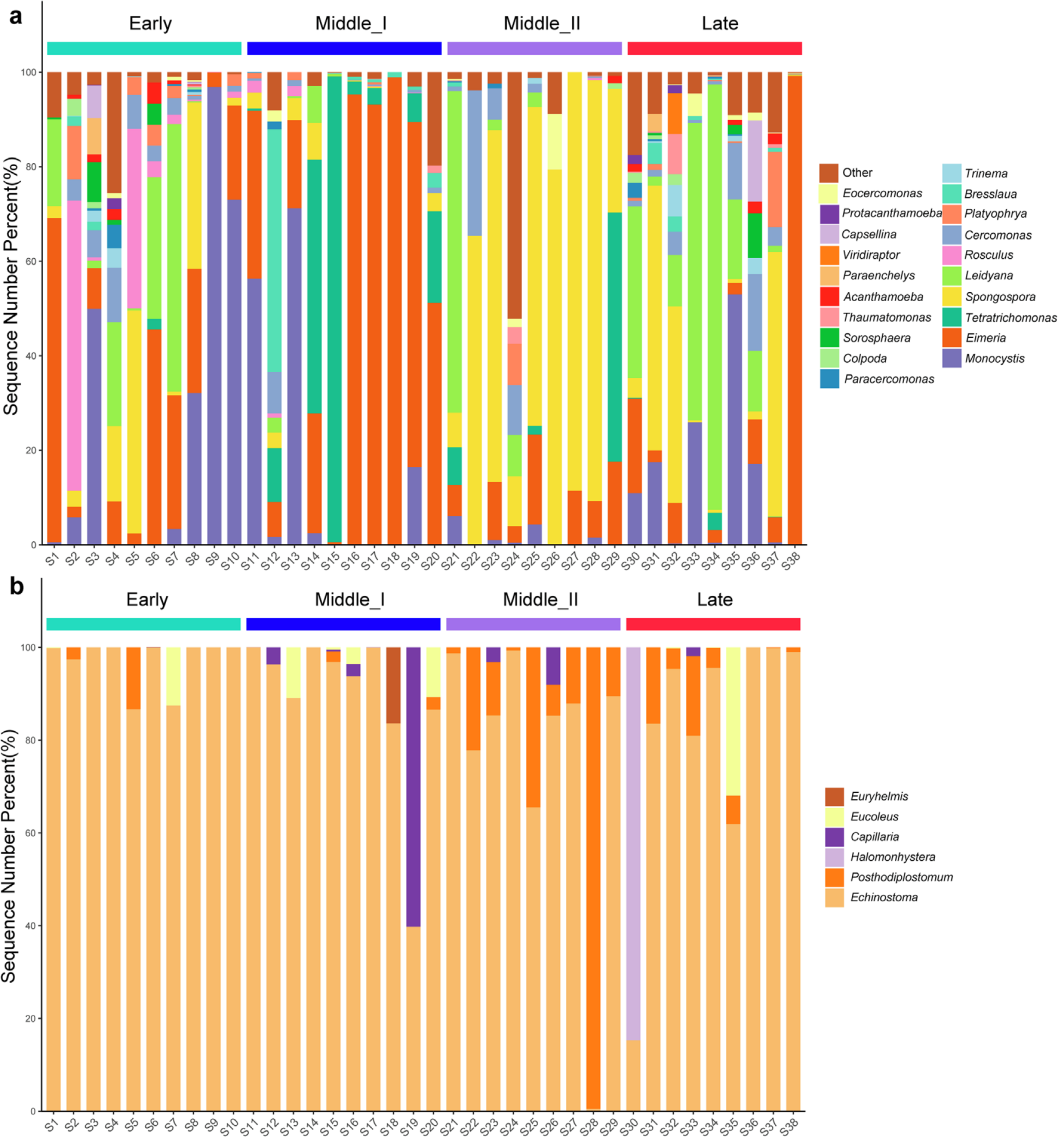
Composition of protozoa and helminth in *Grus nigricollis*. Top 20 genera with the most abundant genera of protozoa (a) and all helminths (b) for each sample.

Based on the SILVA database, a total of 243 ASVs were identified as protozoa and belonged to ten phyla: Discosea, Evosea, Tubulinea, Heterolobosea, Fornicata, Parabasalia, Apicomplexa, Endomyxa, Ciliophora, and Cercozoa (Table S2). Furthermore, a total of 147 ASVs were assigned taxonomy at the genus level and further identified as 64 genera of protozoa. The top 20 genera with the highest relative abundance are shown in Figure 1a: most of the genera belonged to Cercozoa (*Eocercomonas* sp., *Capsellina* sp., *Viridiraptor* sp., *Thaumatomonas* sp., *Paracercomonas* sp., *Trinema* sp., *Cercomonas* sp., and *Rosculus* sp.) and Ciliophora (*Paraenchelys* sp., *Colpoda* sp., *Bresslaua* sp., and *Platyophrya* sp.). Compared with protozoa, helminths had lower diversity with 22 ASVs and belonged to Nematoda and Platyhelminthes (Table S2). Furthermore, 19 ASVs were assigned taxonomy at the genus level: *Euryhelmis* sp., *Eucoleus* sp., *Capillaria* sp., *Echinostoma* sp., *Posthodiplostomum* sp., and *Halomonhystera* sp. (Figure 1b).

**Figure 2:**
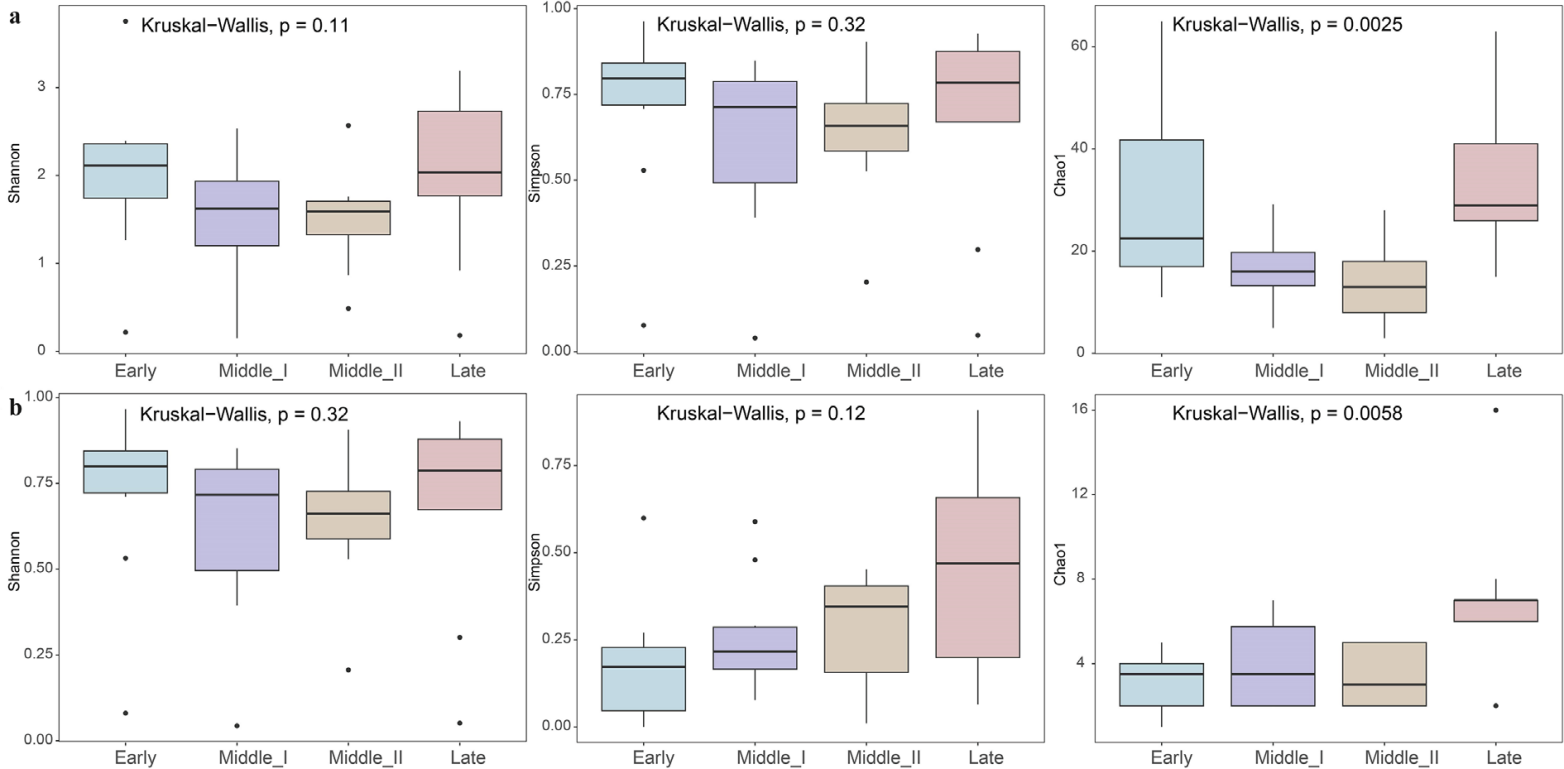
Changes in protozoa (a) and helminth (b) diversity during the wintering period of the black-necked crane, *Grus nigricollis*. Differences in dietary Shannon, Simpson, and Chao1 indices among wintering periods. Values are presented as means ± sd. After a Kruskal–Wallis test, *post hoc* comparisons using the Dunn test with a Benjamini–Hochberg correction indicated a significant difference among wintering periods, as indicated by the different letters (*p* < 0.05).

**Figure 3:**
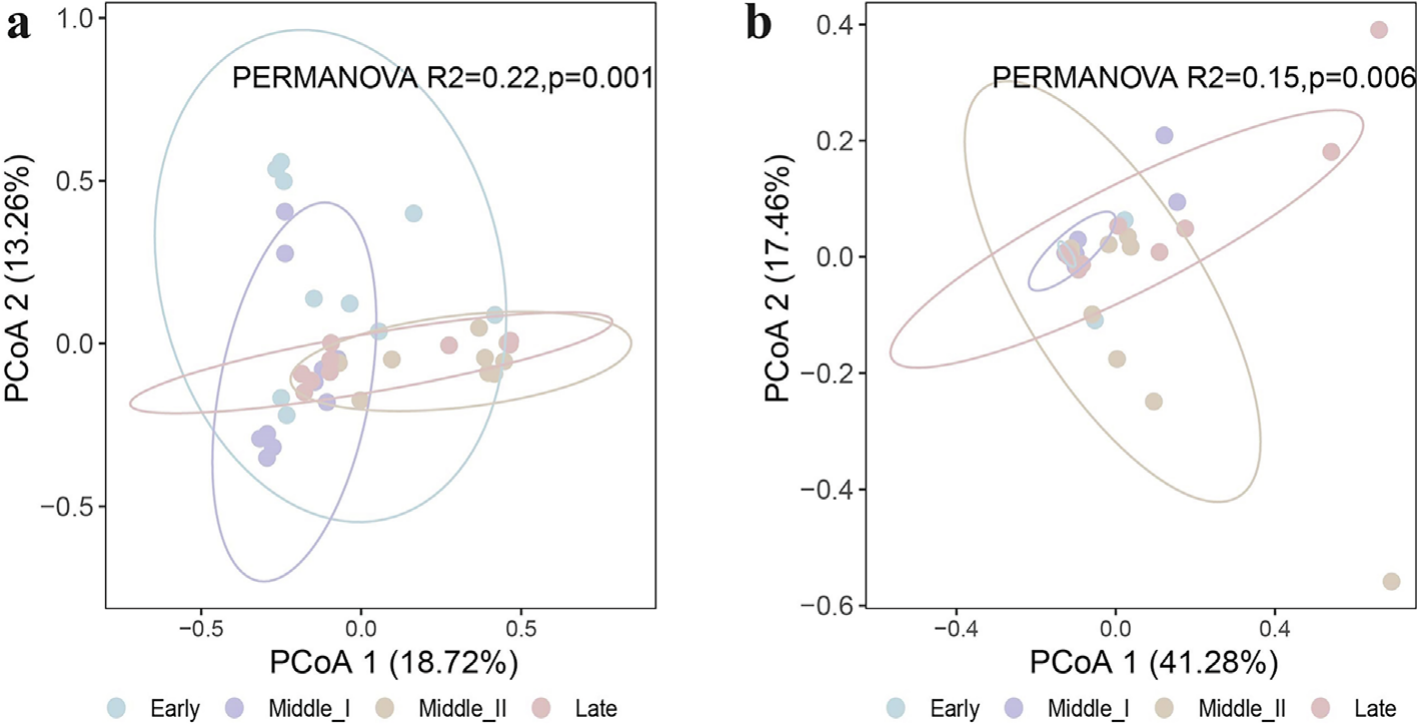
Principal coordinate analysis (PCoA) of protozoa (a) and helminths (b) based on Bray–Curtis distances of the relative abundance of plant amplicon sequence variants (ASVs).

Alpha diversity analysis showed that, except for Chao 1 indices (*p* < 0.05), the Shannon and Simpson indices at the ASV level revealed no significant differences in the protozoa (Figure 2a) or helminths (Figure 2b) among different wintering periods. Principal coordinate analysis (PCoA) based on Bray–Curtis distance at the ASV level (Figure 3) also revealed no distinct clustering pattern in the structure of protozoa or helminths among different wintering periods, but PERMANOVA analysis indicated significant differences (protozoa: PERMANOVA: *R*^2^ = 0.22, *p* = 0.001; helminths: PERMANOVA: *R*^2^ = 0.15, *p* = 0.006).

### Composition and prevalence of parasites in *G. nigricollis*

Based on the 18S rDNA data, a total of 32 ASVs were identified as nine genera of parasites, then, the genus identity of 31 ASVs was further confirmed by BLAST (Table S3), including three protozoan parasites and five helminths (Table 1). Among the protozoan parasites, *Eimeria* sp. had the highest prevalence (92.1%), followed by *Tetratrichomonas* sp. (36.8%). *Theileria* sp. was only detected in one sample. In the helminth, *Echinostoma* sp. was detected in all the samples, while *Posthodiplostomum* sp. (50.0%) and *Euryhelmis* sp. (7.9%) were identified from nineteen and three samples, respectively. By comparison, the nematode parasites had a lower prevalence than that of trematode (Table 1), as follows: *Eucoleus* sp. (26.3%) and *Halomonhystera* sp. (2.6%).

**Table 1:** Positive rates for 16 genera of FLA and parasites in fecal samples of black-necked cranes.

Genus	Prevalence rate (%)	No. positive			
		Early (*n* = 10)	Middle I (*n* = 10)	Middle II (*n* = 9)	Late (*n* = 9)
FLA					
*Acanthamoeba* sp.	34.2	7	0	1	5
*Protacanthamoeba* sp.	13.2	2	0	0	3
*Vahlkampfia* sp.	2.6	1	0	0	0
*Paravahlkampfia* sp.	2.6	0	0	0	1
*Allovahlkampfia* sp.	7.9	2	0	0	1
*Copromyxa* sp.	2.6	0	0	0	1
*Cercomonas* sp.	81.6	8	8	6	9
*Rosculus* sp.	31.6	8	3	1	0
Protozoa parasite					
*Eimeria* sp.	92.1	10	10	7	8
*Tetratrichomonas* sp.	36.8	0	8	3	3
*Theileria* sp.	2.6	0	0	0	1
Helminth					
*Echinostoma* sp.	100	10	10	9	9
*Posthodiplostomum* sp.	50	2	2	8	7
*Euryhelmis* sp.	7.9	1	2	0	0
*Eucoleus* sp.	26.3	3	4	0	3
*Halomonhystera* sp.	2.6	0	0	0	1

The specific PCRs were performed to further identify the species of *Echinostoma* sp., *Tetratrichomonas* sp., and *Theileria* sp. The positive of *Echinostoma* sp. was confirmed by PCR using the genus-specific primers; however, the sequencing result showed that all the raw sequences had stretches of indistinguishable peaks. Moreover, the detection results of the six zoonotic species of *Echinostoma* sp. using the species-specific primers were negative. In the 14 *Tetratrichomonas* sp. positive samples, the same sequence (PP530895) was obtained and further identified as *T. gallinarum* by BLAST. In the 18S data, *Theileria* sp. was detected from only one sample with a low number of reads; however, the specific PCR detection of *Theileria* sp. showed a negative result.

### Composition of free-living amoeba in *G. nigricollis*

Within the protozoa, eight genera of FLA (Table 1) were found from 33 fecal samples of *G. nigricollis*, including *Acanthamoeba* sp. (34.2%), *Protacanthamoeba* sp. (13.2%), *Vahlkampfia* sp. (2.6%), *Paravahlkampfia* sp. (2.6%), *Allovahlkampfia* sp. (7.9%), *Copromyxa* sp. (2.6%), *Cercomonas* sp. (81.6%), and *Rosculus* sp. (31.6%)*.* The specific PCR was performed to identify the pathogenic *Acanthamoeba* sp., and only one sequence (PP530894) was obtained from the 13 samples, which had 99.7% nucleotide identity with the *Acanthamoeba* ST2 (MZ318355).

### Changes in the relative abundance of parasites and FLA in *G. nigricollis* during the overwintering

**Figure 4:**
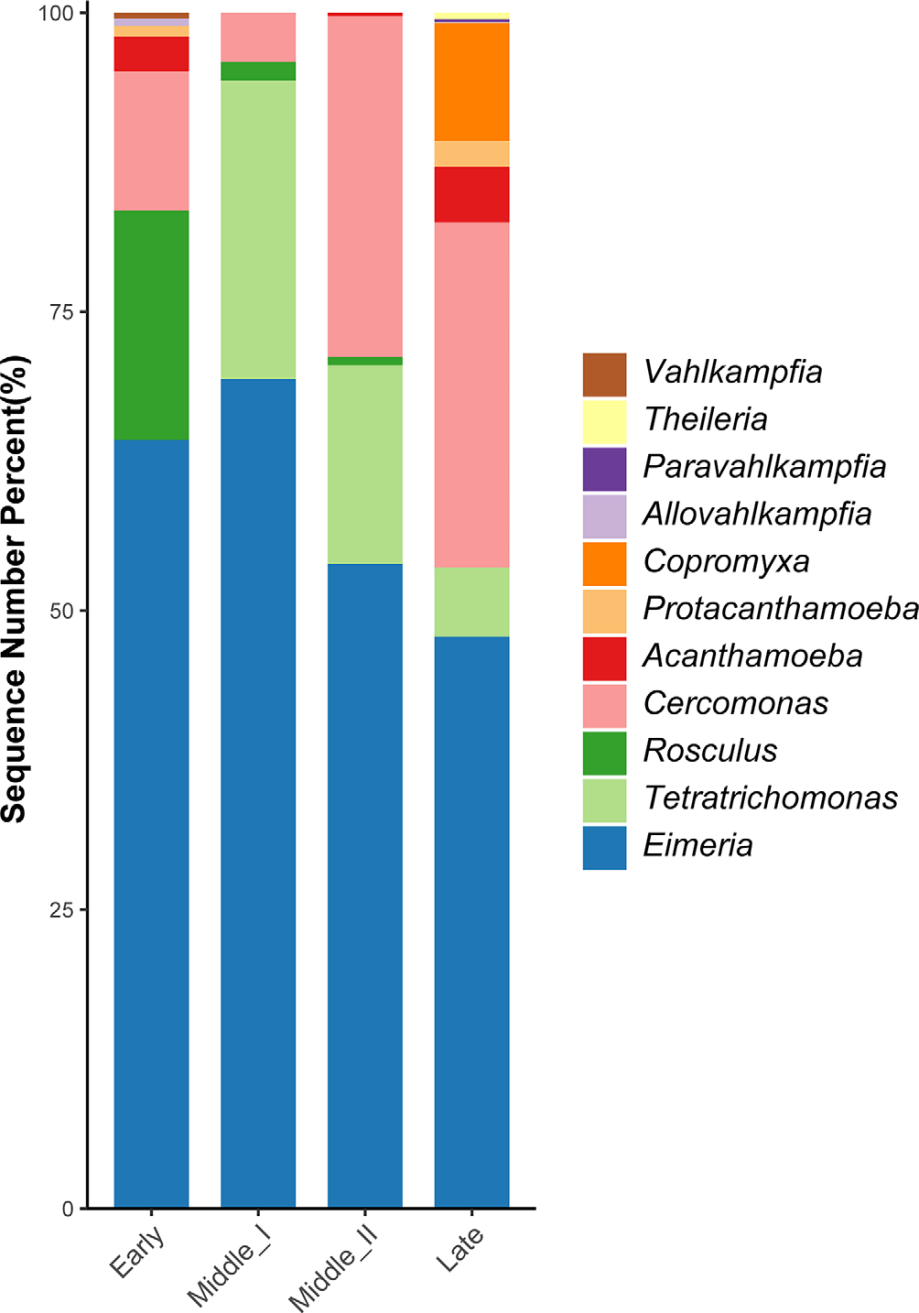
Relative abundance of 11 genera of free-living amoebae and protozoan parasites in *Grus nigricollis* based on four overwintering periods.

The relative abundance based on the four overwintering periods revealed the changes of these focused protozoa in the fecal samples of *G. nigricollis* during overwintering (Figure 4). As the most prevalent protozoan parasite, *Eimeria* sp. had highest relative abundance in all the periods, and its relative abundance decreased from the Middle I to the Late periods (Figure 4). The relative abundance of *Tetratrichomonas* sp. exhibited similar changes with *Eimeria* sp. (Figure 4).

In addition, there was a significant change in the genus diversity of FLA during the overwintering: Early and Late periods both identified six genera; however, the Middle I and Middle II periods only had two and three genera, respectively (Table 1). Furthermore, *Rosculus* sp. had a higher relative abundance in the Early period, but it decreased in the two Middle periods, and none of the samples were positive in the Late period. Meanwhile, *Cercomonas* sp. became the dominant FLA in the Late period with a higher relative abundance than those of other FLA (Figure 4).

## Discussion

In recent years, the metabarcoding technique has become a prevalent method to investigate the diversity of microorganisms in a range of environments (marine, freshwater, soils, etc.) [68, 78], including the intestinal microenvironment [61]. HTS based on the 18S rDNA V9 region has been applied to the detection of parasites in various host animals, such as non-human primates [21], artiodactyls [29], and rodents [6], and in environmental samples, such as wastewater [78]. Our study is the first to use this method to obtain data on the composition of protozoa and helminth in black-necked cranes, and we further focused on the prevalence of parasites and FLA in this near-threatened bird and detected zoonotic or pathogenic species using specific PCRs.

In fecal samples of black-necked cranes collected from Dashanbao, a total of 64 genera of protozoa were identified. Most of the protozoa genera with high relative abundance belonged to the phyla Cercozoa and Ciliophora, which are dominant groups of free-living eukaryotic protozoan in soils and water [16, 24]. Previous studies reported the members of these phyla may be the symbiotic microbes in plants and animals [6, 30, 32, 70]; however, some genera had only been reported in environmental samples (*Platyophrya* sp. [26], *Bresslaua* sp. [9], *Colpoda* sp. [25]). A previous study on the diet of the black-necked cranes in Dashanbao revealed that cranes dig up the soil to find and consume underground food, such as roots or tubers [19]. This foraging behavior may lead black-necked cranes to accidentally ingest free-living protozoa in the soil.

In the protozoa communities, three genera are common internal parasites of vertebrates: *Eimeria* sp., *Tetratrichomonas* sp., and *Theileria* sp. *Eimeria* spp. are obligate intracellular parasites and pathogens of coccidiosis, which is the most significant ubiquitous disease in the chicken industry [12]. In cranes, disseminated visceral coccidiosis caused by *Eimeria* spp. was recognized as a disease entity in captive populations [49]. Infections with *Eimeria* spp. were also reported in wild cranes such as red-crowned cranes [28], white-naped cranes [27], and hooded cranes [44]. A previous study reported the prevalence of *Eimeria* spp. in black-necked cranes in Dashanbao, with three species identified by microscopic observation and a lower prevalence (46.3%) than that in our study (92.1%) [41]. The significant difference in the prevalence rate may be due to different sampling years. Furthermore, the sensitivity of microscopic detection methods is lower than that of molecular detection methods [75], which may lead to a lower observed prevalence rate. Nonetheless, both the results of the present study and the previous study indicate that potential pathogens of coccidiosis are commonly prevalent in black-necked cranes in Dashanbao. Additionally, our study for the first time reported the prevalence of *Tetratrichomonas* sp. in these birds, and *T. gallinarum* was further identified by specific PCR. *Tetratrichomonas gallinarum* infection can cause high mortality rates in poultry [38, 39], but in many cases, it can be asymptomatic or mild. Therefore, further research is needed to investigate the pathogenicity of this parasite to the black-necked cranes. In addition, the 18S rDNA data showed there was only one sample that detected *Theileria* spp. with a low relative abundance; however, the specific PCR result of *Theileria* spp. detection was negative. At present, no research has shown that *Theileria* spp. can infect birds. Considering the low relative abundance observed in the only sample, we speculate that the black-necked crane may not have been infected by *Theileria* parasites.

In addition to protozoan parasites, five genera of helminths were detected in the present study, and trematode parasites had a high positive rate. Most members of trematode have a complex life cycle, infecting gastropod molluscs and fishes as intermediate hosts and vertebrates as the final host, such as *Echinostoma* spp. [11] and *Posthodiplostomum* spp. [1] exhibit a wide distribution and commonly use birds as the final host. Moreover, some *Echinostoma* spp. are known to infect humans [58, 69]. Although our study could not identify the species of *Echinostoma*, the specific PCRs of six zoonotic *Echinostoma* spp. (*E. hortense*, *E. macrorchis*, *E. revolutum*, *E. ilocanum*, *E. cinetorchis*, *E. indoense*) showed negative results. Another trematode, *Euryhelmis* spp. was reported as an intestinal parasite of amphibians [17] and mammals that eat frogs and salamanders [62], but the present study is the first to report this parasite in birds. In the nematode parasites, *Halomonhystera* spp. have been recorded as parasites of crustaceans [52], but they may not be intestinal parasites of black-necked cranes. Unlike *Halomonhystera* spp., another nematode *Eucoleus* spp. are common parasites in birds and are widely distributed in the world [10, 15, 20, 59]. Overall, given that these parasites found in black-necked cranes have a wide host range and their transmission is mainly through the fecal-oral route, we suggest that there is a high risk of cross-transmission between black-necked cranes and other wild animals in Dashanbao, which should be paid greater attention.

In this study, eight genera of protozoa identified in the fecal samples were FLA. FLA are omnipresent in various environments: water (fresh and sea water) and soil [60]; therefore, it is possible that these FLA were incidentally ingested by the cranes, or come from the soil through contact of feces with the environment. Importantly, among the eight FLA identified in this study, *Acanthamoeba* spp. and *Allovahlkampfia* spp. are the causative agents of granulomatous amoebic encephalitis and amoebic keratitis [50]. Moreover, the number of severe and life-threatening infections in humans caused by *Acanthamoeba* spp. has been increasing in recent years [56]. In our study, the genotype of *Acanthamoeba* T2 was further identified. A previous study reported *Acanthamoeba* keratitis and granulomatous amoebic encephalitis caused by T2 [71]. Although its pathogenicity in birds is unknown, it should be of concern due to the threat to human public health. Six other genera of FLA identified in our study were reported in soil and natural or public water [13, 51, 65], of which two genera *Vahlkampfia* and *Paravahlkampfia* produced cytopathology in tissue culture monolayers [64], but there is a lack of evidence to suggest that they are pathogenic in animals.

Concerning changes in the prevalence of FLA in the black-necked cranes during the overwintering period, we observed that the diversity of FLA genera in the Early and Late periods was higher than that in the two Middle groups. Given that the observed FLA may be incidentally ingested by cranes, we suggest that this may be the result of dynamic changes in FLA abundance in the environment. Unlike parasites whose life cycle mainly relies on their hosts, free-living protozoa are directly influenced by environmental factors [3]. Among the parasites identified in our study, the relative abundance of *Eimeria* spp. and *Tetratrichomonas* spp. decreased in the last two periods. However, the observed changes in the relative abundance of the protozoan parasites among the four periods were mainly caused by the decrease in prevalence rates. The abundance of parasites in the environment, which was influenced by environmental factors, may determine the risk of parasite infection in wild animal hosts, hence the prevalence rates of parasites [67]. Importantly, various factors related to the host affect parasite colonization, especially immunoregulation. Meanwhile, immune changes may also affect the abundance of parasites in the host body [80]. In summary, exploring the factors driving the dynamics of parasitic epidemics remains a challenge.

In our study, although 16 genera of FLA and parasites were identified, some noteworthy parasites may be hidden because of the potential primer set bias. A previous study used three PCR primer pairs, targeted the different regions of the 18S rDNA, and obtained complementary taxa of the parasite communities, suggesting that including more than one primer pair could provide additional information for parasite diversity [21]. Furthermore, improving primer design to target particular taxonomic groups of organisms could also increase resolution within particular taxonomic groups [46]. However, gaps in the reference database may also reduce the observed taxonomic group, and the limitation of 18S amplicon sequencing makes it impossible to assign at the species level [37]. These situations pose challenges for the application of high-throughput sequencing technology in parasite detection.

Based on the 18S rDNA data, our study identified helminths and protozoa in the fecal samples of the black-necked cranes. Some of these are known or likely to be parasites of cranes (e.g., coccidia, trichomonad, nematodes, and trematode), while others may be incidentally ingested. Furthermore, the pathogenic FLA *Acanthamoeba* T2 was identified. Therefore, we suggest that more attention should be paid to preventing the transmission of this zoonotic pathogen in Dashanbao. Moreover, long-term monitoring of the prevalence of parasites or other pathogens in the black-necked cranes is of great significance for protecting this near-threatened species.

## Data Availability

The metagenomic data have been deposited in the China National GenBank database (CNGBdb) under project ID CNP0004969 (CNGBdb – China National GeneBank DataBase).

## Supplementary Material

The supplementary material of this article is available at https://www.parasite-journal.org/10.1051/parasite/2024028/olm.

*Supplementary Table S1*. Primers used in specific PCRs. *Supplementary Table S2*. Original classification of protozoa and helminths. *Supplementary Table S3*. BLAST results of the 31 sequences of parasites.
